# The Atmospheric Chemistry of Fluoroacetonitrile and the Characterization of the Major Product, Cyanoformyl Fluoride

**DOI:** 10.3390/molecules30030478

**Published:** 2025-01-22

**Authors:** Ramesh Sapkota, Trang Nguyen, Paul Marshall

**Affiliations:** 1 Department of Chemistry, Rhodes College, 2000 North Parkway, Memphis, TN 38112, USA; sapkotar@rhodes.edu; 2 Department of Chemistry, University of North Texas, 1155 Union Circle #305070, Denton, TX 76203, USA; trangnguyen9@my.unt.edu; 3 Department of Chemistry and Center for Advanced Scientific Computing and Modeling, University of North Texas, 1155 Union Circle #305070, Denton, TX 76203, USA

**Keywords:** kinetics, mechanism, relative rate, cross-section, global warming potential

## Abstract

Fluorinated nitriles have been proposed as low-global-warming-potential substitutes for industrial applications such as plasma etching and as dielectric materials in high-voltage equipment. FT-IR spectroscopy was used to measure the radiative efficiency of CH_2_FCN and its reactivity towards Cl and OH radicals, and to determine products from the Cl reaction. Relative rate experiments yielded rate constants for Cl and OH reactions of (2.1 ± 0.3) × 10^−14^ and (7.0 ± 1.0) × 10^−14^ cm^3^ molecule^−1^ s^−1^, respectively. The estimated atmospheric lifetime of CH_2_FCN with respect to radical attack was estimated to be 0.45 years, which, combined with the radiative efficiency of 0.042 W m^−2^ ppb^−1^, implies a 100-year global warming potential of 20. FCOCN was observed as the only organic product of the Cl-atom reaction in air, consistent with a dominant role for H-abstraction. Absolute infrared cross-sections for FCOCN were determined, to assist future experiments where this molecule may be formed. Quantum calculations at the CBS-APNO//B2PLYP-D3/cc-pVTZ level indicate similar energy barriers to addition and abstraction for OH radical attack, but the looser transition state and greater opportunity for tunneling also favor abstraction in this case.

## 1. Introduction

The Kigali Amendment to the Montreal Protocol [1] regulates environmentally persistent compounds that strongly absorb outgoing infrared (IR) radiation from the Earth’s surface, in order to mitigate climate change. With this motivation, there are efforts underway to replace industrial chemicals that possess high global warming potentials (GWPs). The GWP metric of a compound indicates the integrated amount of IR absorption by this compound, if introduced into the atmosphere, over a specified time interval (100 years for the GWP_100_) on a mass basis, relative to carbon dioxide [2]. Perfluoroalkanes have been widely used as reagents in the plasma etching of wafers for microelectronic manufacture, and an example compound, C_2_F_6_, has a GWP_100_ of, ca., 13,000 [3]. The introduction of reactive functional groups that shorten the lifetime in the atmosphere diminish the GWP. The quantification of this metric requires assessment of the IR absorption in the atmospherically relevant region, combined with the determination of atmospheric lifetime. This lifetime is typically controlled by the rate of consumption by naturally occurring hydroxyl radicals (OH) along with a smaller contribution by atomic chlorine (Cl). Fluoroacetonitrile, CH_2_FCN, and other nitriles have been proposed as plasma etchants [4], but presently, the information needed to determine the GWP of CH_2_FCN is unavailable. Accordingly, we present its measured IR spectrum and kinetics with OH and Cl to determine this GWP. We also compare these results with quantum chemistry analysis to assess the usefulness of theoretical analysis of this and similar compounds.

Fluorinated nitriles exhibit favorable dielectric constants and have been suggested [5] as substitutes for sulfur hexafluoride, SF_6_ (GWP_100_ ~25,000 [3]), used to prevent arcing in high-voltage equipment. This has motivated recent studies of perfluoronitrile chemistry [5,6,7,8]. We speculate that the GWP of such substitutes, which lie in the range of 200–2000 [7], could be lowered further by the inclusion of C-H bonds. We note that this could allow the formation of cyanoformyl fluoride, FCOCN, as an atmospheric degradation product. This molecule was first prepared in 1960 by Tullock and Coffman [9], and in 1991, the complete IR spectrum was obtained and analyzed by Balfour et al. [10]. However, IR absorption cross-sections remain unavailable but are central to the determination of the yield of FCOCN. We chose CH_2_FCN in part as a molecule likely to form FCOCN in large quantities during radical-initiated oxidation. We present a photochemical synthesis of FCOCN, which avoids the original use [9] of toxic phosgene and hydrogen cyanide, and quantify the IR absorbance of the pure compound. These data can then be used to evaluate FCOCN formation from other species.

When nitriles react with radicals, there is the possibility for two reaction pathways: the abstraction of an H-atom or addition to the CN group, as exemplified by acetonitrile, CH_3_CN. For OH, both pathways are important at ambient conditions, while Cl proceeds by abstraction [11]. There are no prior measurements on CH_2_FCN for comparison, but we note that CH_2_FCN is isoelectronic with hydroxyacetonitrile, HOCH_2_CN, a species which has recently been detected in interstellar nebulae [12] and in smoke plumes from wildfires [13]. Its atmospheric reactions with OH were evaluated computationally [14] and are compared with the measurements here. For OH + CH_2_FCN, we predict that H-abstraction dominates, based on ab initio computations for the reaction pathways. The GWP_100_ is estimated as 20, which is indeed an order of magnitude smaller than for perfluoronitriles.

## 2. Results

### 2.1. Infrared Spectra

**Figure 1:**
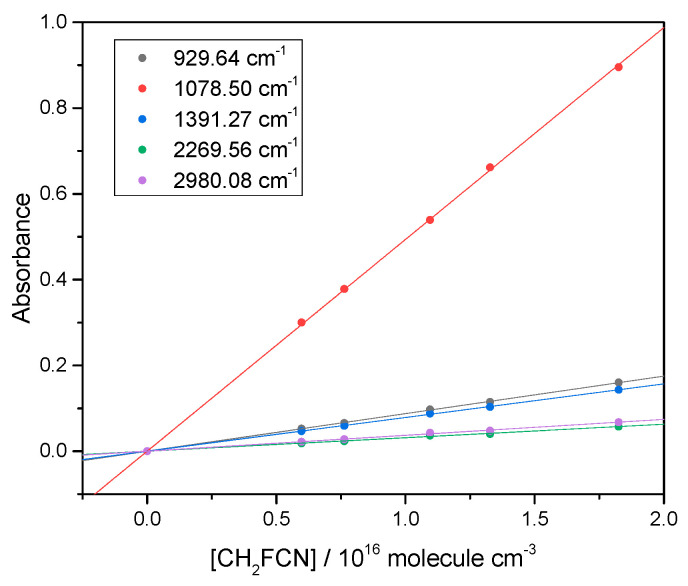
Beer–Lambert plots for peaks in the CH_2_FCN IR spectrum, to check that base e absorbance is proportional to concentration. Each line corresponds to a different band.

We measured the CH_2_FCN IR spectrum and validated the Beer–Lambert law (Section 4.1) applied at the band peaks. This is demonstrated in Figure 1.

**Figure 2:**
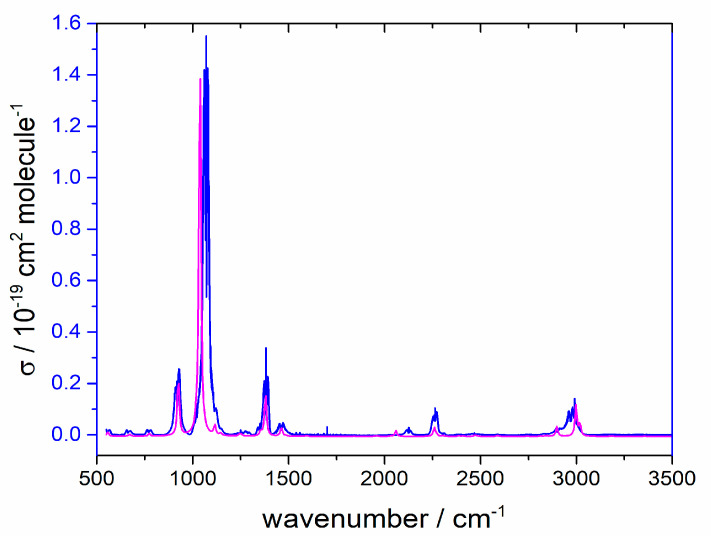
Measured base e cross-section for CH_2_FCN at 292 K (blue line) and computed spectrum with an arbitrary vertical scale (magenta line).

Absolute cross-sections are plotted as a function of wavenumber in Figure 2. Also shown there are anharmonic computed vibrational transitions, which reproduce the band centers well and account even for the weak overtones and combination peaks. The overlap between this spectrum and the radiative forcing efficiency (the distribution of the Earth’s emitted IR, some of which is intercepted by greenhouse gases) evaluated as outlined by Hodnebrog et al. [2] yields a radiative efficiency (RE) of CH_2_FCN as 0.042 W m^−2^ ppb^−1^. Two peaks which lie within the 500–1500 cm^−1^ region mainly contribute to this RE, the strong absorbance at 1070 cm^−1^ from C-F stretching and the moderate absorbance at 918 cm^−1^ due to C-C stretching. The computed spectrum shows two weak peaks below the ~550 cm^−1^ instrumental cutoff, which would contribute less than 1% to the RE.

**Figure 3:**
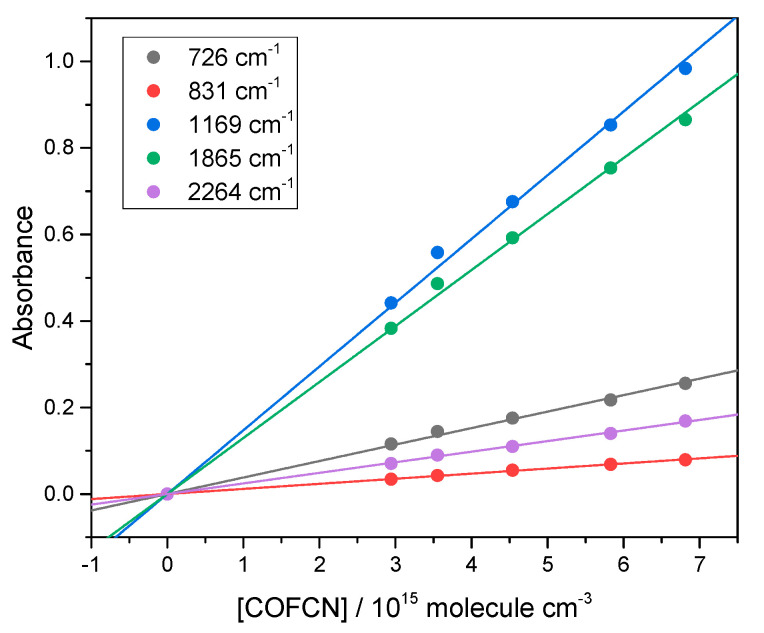
Beer–Lambert plots at band peaks for FCOCN demonstrating linearity.

**Figure 4:**
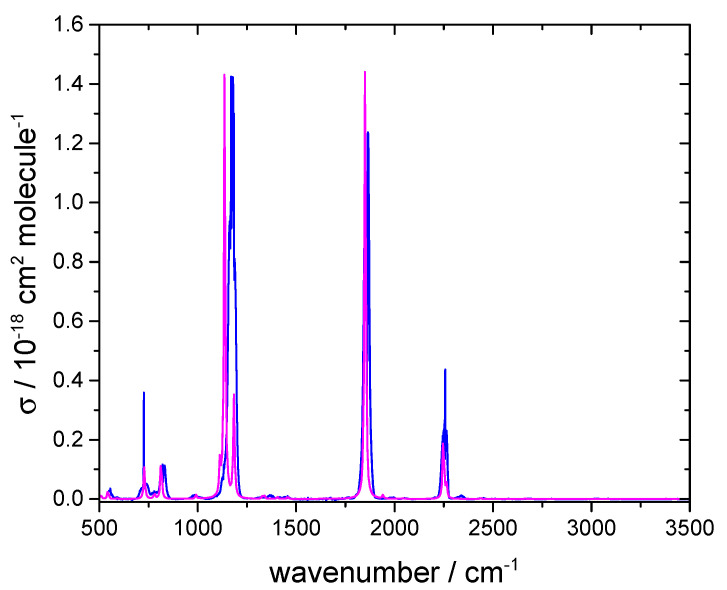
Measured base e cross-section for FCOCN at 292 K (blue line) and computed spectrum with an arbitrary vertical scale (magenta line).

Following its synthesis, we obtained the IR spectrum of FCOCN. The check for proportionality between absorbance *A* and concentration is shown in Figure 3, and the cross-sections are plotted in Figure 4.

Again, the simulated IR spectrum is in good agreement with the measured spectrum and indicates that there are no major impurities causing unassigned bands. The band observed at 2255 cm^−1^ corresponds to C≡N stretching, while the band at 1869 cm^−1^ corresponds to C=O stretching. Experimental and calculated band centers match to within 2 cm^−1^. The bands in the fingerprint region also align closely between the two spectra. We employed the carbonyl band for quantifying FCOCN, with an integrated band strength (base e) of 3.59 ×10^−17^ cm molecule^−1^ over 1862–1880 cm^−1^. This is comparable to 4.6 ×10^−17^ cm molecule^−1^ for carbonyl stretching in COF_2_ [15].

Table S1 gives a detailed list of the measured σ(CH_2_FCN) values as a function of wavenumber, and Table S2 provides the data for σ(FCOCN).

### 2.2. Kinetic Measurements

The rate coefficient *k*_1_ for the reaction

Cl + CH_2_FCN → products (1)

was measured relative to *k*_2_ for

**Figure 5:**
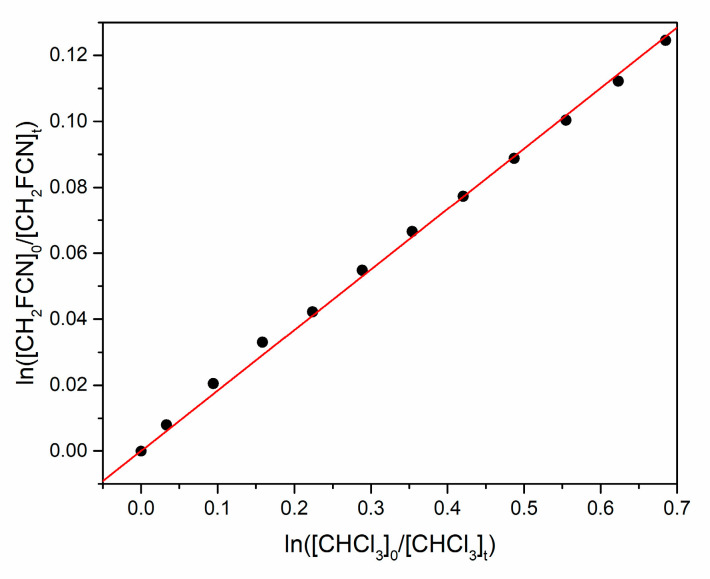
Relative rate plot for simultaneous loss of CH_2_FCN and CHCl_3_ by reaction with atomic Cl.

Cl + CHCl_3_ → products (2)

by monitoring the consumption of both organic reactants using their IR bands at 1090–1045 and 1209–1231 cm^−1^, respectively. Initial checks showed that, without UV irradiation, there was no observable loss of reactants. Figure 5 is an example plot of the concentration data, whose slope equals the ratio *k*_1_/*k*_2_. Table 1 summarizes the partial pressures *p* for four experiments (1 torr = 1.333 mbar) at 292 K, at a total pressure of 750 torr made up with Ar bath gas.

**Figure 6:**
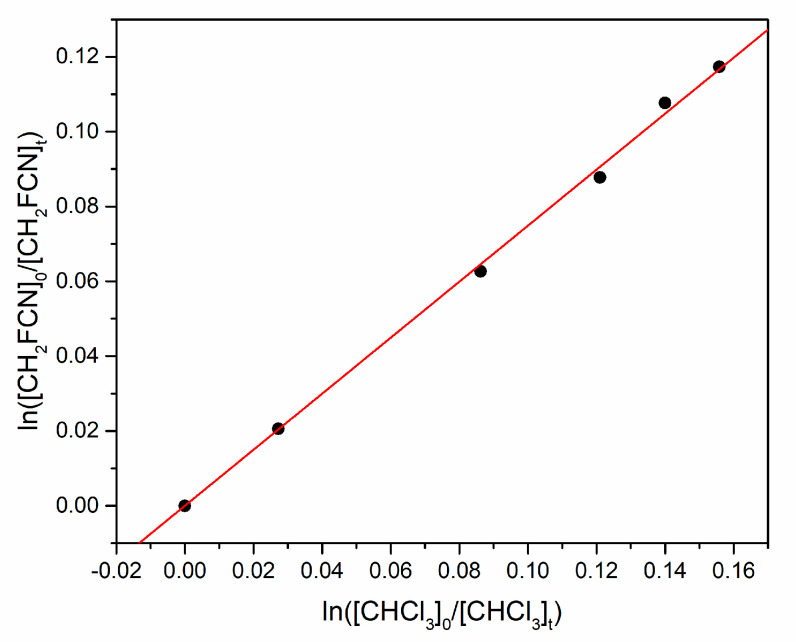
Relative rate plot for simultaneous loss of CH_2_FCN and CHCl_3_ by reaction with OH.

The uncertainty quoted in the rate coefficient ratio is the 1σ statistical uncertainty in the slopes of plots like Figure 5. The JPL critical evaluation recommends *k*_2_ = (1.11 ± 0.17) × 10^−13^ cm^3^ molecule^−1^ s^−1^ at 292 K, with 1σ uncertainty [16]. Thus, our best estimate of *k*_1_ is (2.1 ± 0.3) × 10^−14^ cm^3^ molecule^−1^ s^−1^. The linearity of plots like Figure 5 and Figure 6 indicates that there is no significant secondary chemistry that consumes the organic reagents.

Similarly, the rate coefficient *k*_3_ for the reaction

OH + CH_2_FCN → products (3)

was measured relative to *k*_4_ for

OH + CHCl_3_ → products (4)

by monitoring the consumption of organic reactants by their IR bands at 1398–1365 and 1212–1230 cm^−1^, respectively. Figure 6 is an example plot of the concentration data, whose slope equals the ratio *k*_3_/*k*_4_. Table 2 summarizes the experimental conditions for three experiments (1 torr = 1.333 mbar) at 291 K and 750 torr total pressure made up with Ar.

The JPL evaluation recommends *k*_4_ = (9.3 ± 1.4) × 10^−14^ cm^3^ molecule^−1^ s^−1^ at 291 K, with 1σ uncertainty [16]. Thus, our best estimate of *k*_3_ is (7.0 ± 1.0) × 10^−14^ cm^3^ molecule^−1^ s^−1^.

### 2.3. Product Studies

**Figure 7:**
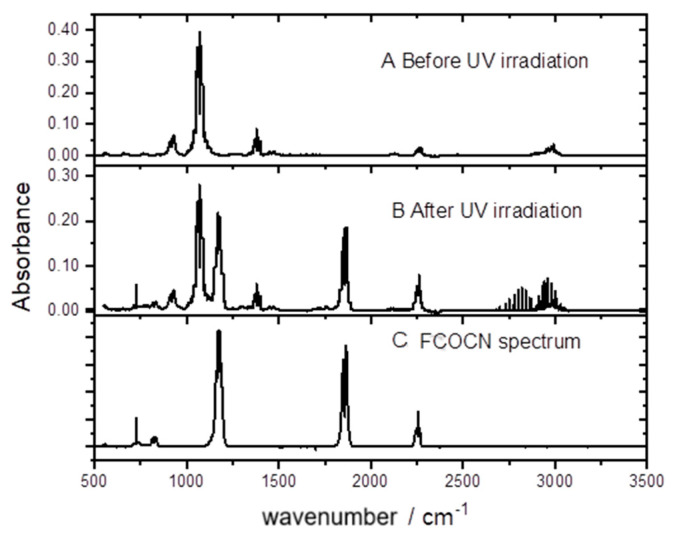
Spectra of an irradiated CH_2_FCN/Cl_2_/air mixture.

In order to gain insight into the likely atmospheric chemistry of CH_2_FCN following initial radical attack, experiments were performed in zero-air bath gas with Cl_2_ as the radical source and CH_2_FCN as the only organic reagent. The growth of products was monitored via their IR spectra. An example is shown in Figure 7.

**Figure 8:**
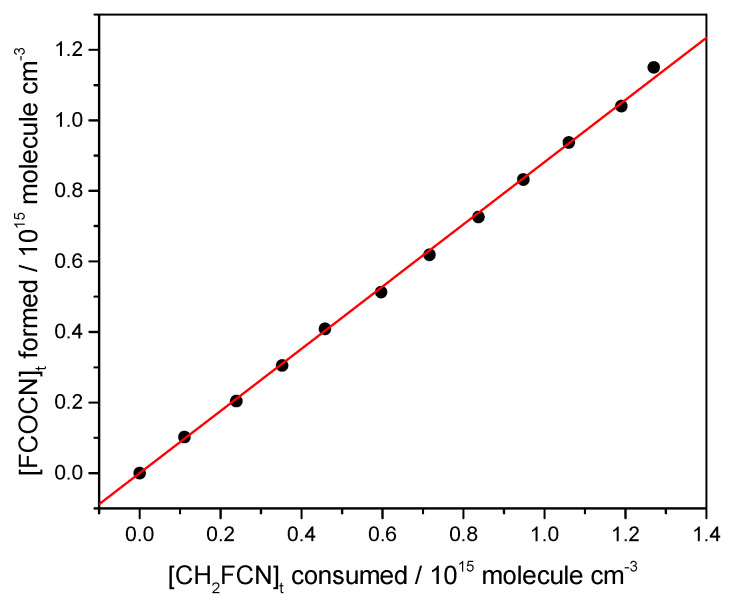
Yield of FCOCN from an irradiated CH_2_FCN/Cl_2_/air mixture, starting with 0.24 torr of CH_2_FCN and 4.1 torr of Cl_2_ made up to 760 torr with zero air.

It may be seen that FCOCN is a major product. This was quantified by plotting the concentration of FCOCN formed, determined via the carbonyl band strength, against the concentration of CH_2_FCN consumed. A correction was included for a dark loss of CH_2_FCN noted in these experiments of 0.5% per minute. The results are shown in Figure 8, whose slope gives a yield of 88%. HCl is also formed and accounts for the line spectrum in the 2700–3100 cm^−1^ region. We attempted to measure its yield and obtained 68%, but because HCl readily absorbs on surfaces, this is a lower bound.

### 2.4. Pathways for Reaction with Hydroxyl

**Figure 9:**
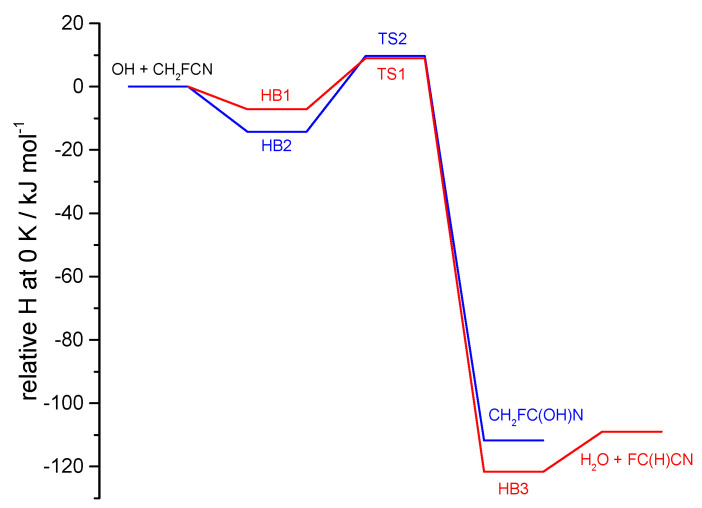
CBS-APNO//B2PLYP-D3/cc-pVTZ energies including zero-point vibrational energy along the reaction paths for OH + CH_2_FCN. The H-abstraction channel is in red, and the addition channel is in blue.

Quantum calculations were carried out to explore the addition and abstraction reaction pathways for OH with CH_2_FCN (Reaction (3)). The geometries of reactants, intermediates, transition states, and products are provided in Table S3. Figure 9 compares the relative enthalpies at 0 K along these two pathways, which both involve moderately bound pre-reactive complexes before distinct energy barriers.

For the abstraction reaction, there is weak (6.3 kJ mol^−1^) hydrogen bonding between the H of the incoming OH radical and the F atom in the complex HB1, before passage over a 9.8 kJ mol^−1^ barrier (relative to the reactants) to form H_2_O + FC(H)CN, via a hydrogen-bonded complex HB3 in the exit channel. For the addition reaction, there is a 13.6 kJ mol^−1^ hydrogen-bonded complex HB2 in the entrance channel, before a barrier of 10.5 kJ mol^−1^ on the way to formation of the CH_2_FC(OH)N addition product, where a new C-O bond is created.

## 3. Discussion

The high yields of FCOCN observed indicate that the abstraction channel dominates the Cl-atom kinetics (Reaction (1)). A plausible mechanism is that initially formed FC(H)CN reacts with O_2_ to create peroxy radicals, FC(H)(CN)OO. In the atmosphere, these could react with NO to yield alkoxy species, FC(H)(CN)O. The same species can be formed in the laboratory by the peroxy self-reaction ROO + ROO → RO + RO + O_2_, which is favored at the high radical concentrations used in experiments (high compared to the atmosphere). The C-H bond is weak in such alkoxy radicals, because the loss of H· is accompanied by C-O· rearrangement to C=O, so H is readily removed by collision with O_2_ to make HO_2_. This leads to the observed FCOCN product. In fact, Figure 7 shows no other IR-active product (except HCl, the other H-abstraction product). Guo et al. [7] argued that addition would ultimately lead to the creation of nitrogen oxides, but none were observed here. We speculate that the yield of FCOCN is essentially 1, and that the measurement of 0.88 (Figure 8) might indicate some loss of FCOCN on the walls of the IR cell. We suggest that the yield of FCOCN can be represented as 0.9 ± 0.1.

For Reaction (3) with hydroxyl, the reaction profiles in Figure 9 show that the transition state (TS) energies for the competing pathways are essentially equal (to 0.7 kJ mol^−1^, well within the uncertainty of such calculations which may be several kJ mol^−1^). There are two factors that will favor abstraction over addition. One is quantum mechanical tunneling, where the light H-atom transfer has an imaginary frequency of 1063 i cm^−1^ vs. 530 i cm^−1^ for C-O bond formation. The second factor is that the addition TS is tighter than for abstraction, with an entropy at 298 K smaller by about 16 J K^−1^ mol^−1^. This reflects the alignment of OH and CN groups in the TS, which are held parallel by dipole forces, so the barriers to torsion of the OH groups in the TSs are smaller for abstraction (4.6 kJ mol^−1^) than for addition (31 kJ mol^−1^). This entropy difference influences the pre-exponential factors, as seen below. Transition state theory estimates of abstraction and addition rate constants at 291 K are 9.3 × 10^−14^ and 8.0 × 10^−15^ cm^3^ molecule^−1^ s^−1^, respectively, consistent with these interpretations and suggesting a branching ratio of about 0.9 for abstraction by OH. The total rate constant is, ca., 40% larger than observed, which might arise from an underestimation of barrier heights by ~0.9 kJ mol^−1^, within the computational uncertainty. The computed Arrhenius expressions near room temperature are 2.3 × 10^−12^ exp(−7.7 kJ mol^−1^/RT) cm^3^ molecule^−1^ s^−1^ and 5.5 × 10^−13^ exp(−10.2 kJ mol^−1^/RT) cm^3^ molecule^−1^ s^−1^ for TS1 and TS2, respectively. The activation energy difference is influenced in part by the greater tunneling at TS1.

We therefore expect Reaction (3) to be similar to the OH reaction with HOCH_2_CN, where abstraction was also proposed as the main pathway [14], for similar reasons, even though, in that case, the abstraction barrier was *higher* than for addition by ~4 kJ mol^−1^. The HOCH_2_CN abstraction barrier was 5 kJ mol^−1^ lower than that proposed here for CH_2_FCN, which largely accounts for k_3_ being about a factor of 4 smaller than for the predicted analogous abstraction from HOCH_2_CN.

We now consider the tropospheric lifetime of CH_2_FCN, τ, with respect to consumption by Cl and OH radicals. Given the average concentrations [Cl] = 3 × 10^4^ molecule cm^−3^ [17] and [OH] = 1 × 10^6^ molecule cm^−3^ [18], and that *k*_3_ ≈ 3 *k*_1_, the rate of loss via the Cl reaction is negligible, about 1% of that via OH. We also neglect minor stratospheric losses and employ [3]

1/τ = *k*_3_[OH] (5)

to obtain τ = 0.45 years for the troposphere. This lifetime can be combined [2] with the radiative efficiency of 0.042 W m^−2^ ppb^−1^ to estimate the 100-year global warming potential as GWP_100_ = 20. This is small enough to make CH_2_FCN a relatively benign industrial chemical, from the climate change perspective. The incorporation of C-H bonds has shortened the lifetime, compared to perfluoronitriles, by an order of magnitude or more.

## 4. Materials and Methods

### 4.1. Materials

The reagents used were CH_2_FCN (Aldrich, St. Louis, MO, USA, 98% purity), CHCl_3_ (Mallinckrodt, St. Louis, MO, USA, 99.8%), and Cl_2_ (Matheson, Irving, TX, USA, 99.5%), which we purified by freeze-pump-thaw cycles with liquid nitrogen. H_2_ (MG Industries, Valley Forge, PA, USA, UHP grade), O_2_, and Ar (Air Liquide, Pasadena, TX, USA, >99.99% purity) and zero air (20% O_2_/80% N_2_, Airgas, Radnor, PA, USA, industrial grade) were used directly from their cylinders. Ozone was synthesized by passing pure O_2_ through an ozone generator (A2Z Ozone, Louisville, KY, USA) and separating the O_3_ from O_2_ in a trap filled with silica gel and cooled with an acetone/dry ice slush bath.

Cyanoformyl fluoride, FCOCN, was photochemically synthesized from a mixture of 5 torr of CH_2_FCN, 8 torr of Cl_2_, and 250 torr of zero air in a glass bulb exposed to 365 nm UV light. Small samples were taken from the bulb and analyzed by FT-IR spectroscopy to check for remaining traces of CH_2_FCN. UV exposure continued until the CH_2_FCN was consumed, and then the mixture was passed through a liquid nitrogen-cooled trap (77 K) which condensed the FCOCN, while the uncondensed air was removed through pumping. The liquid nitrogen trap was then replaced with an ethanol slush bath at 157 K. This temperature retained FCOCN and HCN in the trap, while more volatile impurities including excess Cl_2_ were removed through multiple freeze–pump–thaw cycles. IR analysis revealed 2.5% HCN, based on a 3220–3384 cm^−1^ band strength of 2.55 × 10^17^ cm molecule^−1^, obtained from the PNNL database [15]. To verify that there was no Cl_2_ present, H_2_ was added to a sample and, after UV irradiation, no HCl was detected.

Five known pressures of FCOCN up to 0.2 torr, corrected for the HCN component, were made up to a final pressure of 750 torr with Ar at 292 K and sent to the FT-IR instrument. Adherence to the Beer–Lambert law was verified for the major peaks. The concentration, absorbance, and path length data were incorporated into Equation (6):

σ = 2.3026 *A*/(*c* ℓ) (6)

where, with the path length ℓ in cm and *c* in molecule cm^−3^, the base e cross-section σ is obtained in cm^2^ molecule^−1^. We determine σ at every discrete frequency in the absorption spectrum, from the slope of a linear plot of measured *A* vs. the values of *c*. The same procedure was employed to evaluate the cross-sections of CH_2_FCN.

### 4.2. Experimental Methods

The apparatus and methodology have been described previously [19]. Briefly, reagents are diluted in bath gas to 750 torr of pressure in a quartz multipass IR cell (path length ℓ = 240 cm) at room temperature. Sequential FT-IR spectra are taken at 1 cm^−1^ resolution over time. Radicals are created by continuous in situ UV photolysis of Cl_2_ at 365 nm for atomic Cl, or of O_3_/H_2_ mixtures at 254 nm, to create OH from O(^1^D) reaction with excess H_2_. Cl atoms were used for product formation studies with zero-air bath gas to simulate atmospheric conditions. Cl and OH were used in relative rate studies, where the consumption of CH_2_FCN (A) and an added reference compound, CHCl_3_ (B), was monitored. The rate coefficients *k*_i_ are related via [20]

(7) $$ln\left(\frac{{\left[CH_{2}FCN\right]}_{0}}{{\left[CH_{2}FCN\right]}_{t}}\right)=\frac{k_{A}}{k_{B}}\;ln\left(\frac{{\left[CH{Cl}_{3}\right]}_{0}}{{\left[CH{Cl}_{3}\right]}_{t}}\right).$$

The quotients are the ratio of the initial species concentration to its later value at a time *t*. With *k*_B_ known, *k*_A_ can be obtained.

### 4.3. Computational Methods

IR spectra are simulated with second-order vibrational perturbation theory [21], as implemented in the Gaussian 16 code, rev A.03 [22]. The incorporation of anharmonicity avoids the empirical scaling of harmonic frequencies, and overtones and combination bands are included. Quantum calculations are made with the N07D basis set, designed for this task [21,23], and used with the B2PLYP density functional [24,25]. The computed vibrational spectral lines are then artificially broadened with Lorentzian functions (16 cm^−1^ FWHM) as a simple representation of the rotational structure which is not generally resolved for the polyatomic molecules in these experiments.

For potential energies along the OH + CH_2_FCN reaction paths, the geometries and harmonic frequencies (scaled as in [14] for zero-point vibrational energy) of stationary points were evaluated with B2PLYP-D3/cc-pVTZ density functional theory [24,26,27], followed by single-point energy evaluations with the CBS-APNO approximation to coupled cluster theory extrapolated to the infinite basis set limit [28]. The OH energy was lowered empirically by 0.83 kJ mol^−1^ to correct for its spin–orbit splitting.

Canonical transition state theory

(8) $$k=\kappa \;m_{TS}\;\frac{k_{B}T}{h}\;\frac{Q_{TS}}{Q_{OH}Q_{{CH}_{2}FCN}}exp\left(-\frac{E_{0}}{RT}\right)$$

with an Eckart correction *κ* for quantum mechanical tunneling, was applied as detailed in a prior study [14]. Internal hydroxyl rotors in the transition states for abstraction and addition were characterized through explicit analysis of the one-dimensional torsional potentials; otherwise, the partition functions Q were evaluated with the rigid-rotor harmonic-oscillator approximation. *m_TS_* is the number of optical isomers for the transition state, and E_0_ is the 0 K energy difference between the transition state and the reactants, including zero-point vibrational energy. The rate constants were evaluated with the MultiWell program [29].

## Figures and Tables

**Table 1:** Conditions for relative rate measurements of Cl + CH_2_FCN.

*p* CH_2_FCN/Torr	*p* CHCl_3_/Torr	*p* Cl_2_/Torr	*k*_1_/*k*_2_
0.44	0.44	1.73	0.18 ± 0.01
0.34	0.34	1.23	0.19 ± 0.01
0.24	0.24	1.03	0.20 ± 0.01
0.17	0.17	1.52	0.20 ± 0.01

**Table 2:** Conditions for relative rate measurements of OH + CH_2_FCN.

*p* CH_2_FCN/Torr	*p* CHCl_3_/Torr	*p* O_3_/Torr	*p* H_2_/Torr	*k*_3_/*k*_4_
0.43	0.43	5.33	30.28	0.75 ± 0.01
0.34	0.34	3.85	21.88	0.75 ± 0.01
0.16	0.16	2.04	11.56	0.75 ± 0.01

## Data Availability

The original contributions presented in this study are included in the article/Supplementary Materials. Further inquiries can be directed to the corresponding author.

## Supplementary Materials

The following supporting information can be downloaded at https://www.mdpi.com/article/10.3390/molecules30030478/s1. Table S1: IR cross-sections of CH_2_FCN as a function of wavenumber; Table S2: IR cross-sections of FCOCN as a function of wavenumber; Table S3: Geometries of stationary points for OH reactions with CH_2_FCN.
