# Supplementary material for: The Atmospheric Chemistry of Fluoroacetonitrile and the Characterization of the Major Product, Cyanoformyl Fluoride
Source: Molecules. 2025 Jan 22;30(3):478. doi: 10.3390/molecules30030478 (PMC11820647; doi:10.3390/molecules30030478)
Supplement: Supplementary file 1 [file molecules-30-00478-s001.zip › Table S3 - Computed OH+CH2FCN geometries.pdf]

B2PLYP-D3/cc-pVTZ geometries

CH2FCN

6	0.629390000	0.516245000	0.000000000
9	-0.329718000	1.504671000	0.000000000
1	1.248107000	0.623739000	0.889086000
6	0.000000000	-0.807341000	0.000000000
1	1.248107000	0.623739000	-0.889086000
7	-0.472156000	-1.863278000	0.000000000

OH

8	0.000000000	0.000000000	0.107864000
1	0.000000000	0.000000000	-0.862909000

HB1

6	-0.255688000	0.390131000	0.602386000
9	0.428577000	1.018074000	-0.431030000
1	-0.494292000	1.135208000	1.357737000
6	-1.486587000	-0.211906000	0.090162000
1	0.392394000	-0.376465000	1.020454000
7	-2.464469000	-0.696333000	-0.293240000
1	2.299957000	-0.073978000	-0.567807000
8	2.706210000	-0.755306000	-0.004214000

TS1 abstraction

6	0.030117000	0.407536000	0.478865000
9	0.262572000	1.510262000	-0.291550000
1	0.040852000	0.670595000	1.534220000
6	-1.188305000	-0.275809000	0.086235000
1	0.933178000	-0.347312000	0.329707000
7	-2.147504000	-0.848597000	-0.218364000
1	2.258779000	-0.544599000	-0.847385000
8	2.048213000	-1.027653000	-0.031831000

Product complex

6	0.723156000	-0.515163000	0.007614000
9	2.026524000	-0.770440000	-0.005178000
6	0.303534000	0.809954000	-0.004523000
1	0.049966000	-1.351815000	0.035958000
7	-0.093385000	1.905937000	-0.010918000
1	-2.670435000	-0.560195000	-0.827933000
8	-2.335775000	-0.786207000	0.043532000
1	-2.438499000	0.025317000	0.548201000

CHFCN

6	0.000000000	0.754919000	0.000000000
9	1.324418000	0.841178000	0.000000000
1	-0.536878000	1.686035000	0.000000000
6	-0.591006000	-0.501283000	0.000000000
7	-1.119550000	-1.539779000	0.000000000

H2O

1	0.000000000	0.757192000	-0.471394000
8	0.000000000	0.000000000	0.117848000
1	0.000000000	-0.757192000	-0.471394000

HB2

6	-0.782301000	-0.553247000	0.000001000
9	-2.120863000	-0.228126000	-0.000017000
1	-0.550956000	-1.137502000	-0.887544000
6	0.035651000	0.664788000	0.000012000
1	-0.550983000	-1.137504000	0.887552000
7	0.704099000	1.610029000	0.000017000
8	2.124150000	-0.928424000	0.000045000
1	2.747712000	-0.183913000	-0.000415000

TS2 addition

6	0.942386000	-0.319008000	0.513135000
9	1.674360000	0.274557000	-0.484556000
1	0.930400000	0.327207000	1.386000000
6	-0.447289000	-0.549645000	0.065424000
1	1.387165000	-1.282921000	0.752967000
7	-1.408530000	-1.132074000	-0.264727000
8	-1.070627000	1.299874000	0.115829000
1	-1.932659000	1.222146000	-0.322857000

OH adduct

6	0.835616000	-0.438784000	0.444074000
9	1.697677000	0.235100000	-0.397711000
1	0.999435000	-0.083522000	1.461173000
6	-0.589917000	-0.155705000	0.049455000
1	1.028480000	-1.505365000	0.377936000
7	-1.386091000	-1.074593000	-0.267357000
8	-0.907083000	1.162125000	0.104868000
1	-1.821900000	1.265070000	-0.188333000
